# The Associations between Callous-unemotional Traits and Symptoms of Conduct Problems, Hyperactivity and Emotional Problems: A Study of Adolescent Twins Screened for Neurodevelopmental Problems

**DOI:** 10.1007/s10802-018-0439-1

**Published:** 2018-05-05

**Authors:** Marie Claire Saunders, Henrik Anckarsäter, Sebastian Lundström, Clara Hellner, Paul Lichtenstein, Nathalie M. G. Fontaine

**Affiliations:** 1 School of Criminology, University of Montreal, C.P. 6128, Succursale Centre-ville, Montreal, Quebec H3C 3J7 Canada; 2 Centre for Ethics, Law and Mental Health, University of Gothenburg, Gothenburg, Sweden; 3 Gillberg Neuropsychiatry Centre, University of Gothenburg, Gothenburg, Sweden; 4 Centre for Psychiatry Research, Department of Clinical Neuroscience, Karolinska Institutet, Stockholm, Sweden; 5 Stockholm Health Care Services, Stockholm County Council, Stockholm, Sweden; 6 Department of Medical Epidemiology and Biostatistics, Karolinska Institutet, Stockholm, Sweden

**Keywords:** Callous-unemotional traits, Conduct problems, Hyperactivity, Emotional problems, Twin study

## Abstract

Callous-unemotional (CU) traits (e.g., lack of empathy, lack of guilt, shallow affect) are associated with severe and persistent conduct problems in youth. There is evidence showing a substantial genetic correlation between CU traits and conduct problems. The etiological associations between CU traits and other psychopathological symptoms, including symptoms of hyperactivity and emotional problems (such as anxiety and depression symptoms), have been less explored. To examine the etiological associations between CU traits and symptoms of conduct problems, hyperactivity and emotional problems separately through the use of a twin design. Participants were same-sex twin pairs (*n* = 426 twins; 42% female; 43% MZ; age = 15) drawn from the Child and Adolescents Twin Study in Sweden, a longitudinal study of twins born in Sweden. The sample was mainly composed of children who screenpositive on neurodevelopmental problems/mental health problems or at-risk children (i.e., screen-negative children considered to be genetically at-risk siblings). We used self-report measures of CU traits, conduct problems, hyperactivity and emotional problems. Model-fitting analyses were conducted using structural equation modeling. We found a strong positive genetic correlation between CU traits and conduct problems and a moderate genetic correlation between CU traits and hyperactivity. We also found a relatively modest, but significant negative genetic correlation between CU traits and emotional problems. Using a sample of adolescent twins screened for neurodevelopmental problems, we replicated previous findings that showed a strong genetic correlation between CU traits and conduct problems and we extended research by examining further the etiological associations between CU traits and symptoms of hyperactivity and emotional problems.

Callous-unemotional (CU) traits, which include characteristics such as lack of empathy, lack of guilt and shallow affect, are a well-documented temperamental risk factor for severe and persistent conduct problems in youth (Fontaine et al. 2011; Frick et al. 2003b). In addition, CU traits have been identified as a precursor to adult psychopathy (Lynam et al. 2007) and are considered the clinical hallmark of this syndrome (Cleckley 1976; Hare 2003). Evidence for a subset of youth with severe conduct problems distinguished by their high levels of CU traits led to the inclusion of CU traits as a specifier (labeled ‘limited prosocial emotions’) to conduct disorder in the *Diagnostic and Statistical Manual of Mental Disorders – 5th edition* (DSM; American Psychiatric Association 2013).

Research on the development of persistent conduct problems, CU traits and adult psychopathy has also focused on the potential contribution of hyperactivity or attention deficit hyperactivity disorder (ADHD; e.g., Lynam 1996). There is evidence suggesting that conduct problems, CU traits and hyperactivity co-occur. Youth with conduct problems (Fontaine et al. 2008; Nagin and Tremblay 2001) and youth with combined high levels of CU traits and conduct problems (Fontaine et al. 2011; Frick et al. 2003a) are also likely to have high levels of hyperactivity. However, empirical findings suggest that the co-occurrence of conduct problems and hyperactivity does not designate a distinct group of youth at risk of developing psychopathy later on (Barry et al. 2000; Forsman et al. 2007).

While there is evidence suggesting that CU traits are associated with externalizing problems, such as conduct problems (Frick et al. 2014) and that externalizing problems are associated with internalizing problems, such as depression and anxiety (Russo and Beidel 1994), the nature of the association between CU traits and internalizing problems is less clear. Based on theory and clinical work, CU traits are expected to be negatively associated with anxiety (Cleckley 1976). A number of empirical studies support this negative association (Frick et al. 1999; Pardini and Fite 2010). In particular, it has been shown that low levels of fearfulness in youth, a trait closely related to anxiety (Kochanska 1993), may be an important risk factor for the later development of CU traits (Glenn et al. 2007; Waller et al. 2016). However, other studies have reported no significant association between CU traits and anxiety (Loney et al. 2003; Neumann and Pardini 2014). In addition, there are even findings suggesting a positive association between CU traits and anxiety (Essau et al. 2006; Fontaine et al. 2011). However, these findings could be notably explained by the fact that the unique and contrasting contributions of CU traits and conduct problems to anxiety were not explored: conduct problems, when controlling for CU traits, tend to be positively correlated with anxiety, whereas CU traits, when controlling for conduct problems, tend to be negatively correlated with anxiety (Frick and Dickens 2006).

## Etiology of CU Traits, Conduct Problems, Hyperactivity and Emotional Problems

Research based on twin samples has yielded important information concerning the etiology of CU traits, conduct problems, hyperactivity and emotional problems. Findings showed moderate to strong heritability of CU traits in youth, especially in boys (Fontaine et al. 2010), with estimates indicating that 40–78% of the variation in CU traits across the population was due to genetic contributions (Viding et al. 2013; Viding and McCrory 2012). These studies have also suggested that non-shared environmental contributions were important to explain variation in CU traits. On the other hand, shared environmental contributions to CU traits were reported in only a small number of studies (Fontaine et al. 2010; Viding et al. 2007), although they may be especially important for a small subset of girls with stable and high levels of CU traits (Fontaine et al. 2010).

Moderate to strong genetic and non-shared environmental contributions have been found to explain the variation in conduct problems (Forsman et al. 2010; Viding et al. 2007) and emotional problems (Blonigen et al. 2005; Mann et al. 2015) in youth samples. Shared environmental contributions were often modest or not significant. As for hyperactivity in youth samples, high heritability estimates and relatively modest to moderate non-shared environmental estimates were reported (Biederman 2005; Kuntsi and Stevensen 2001).

A number of studies have examined the etiological association between CU traits and conduct problems. Genetic and non-shared environmental correlations were reported, but the strength of these correlations varied across studies (Bezdjian et al. 2011; Blonigen et al. 2005; Larsson et al. 2007; Viding et al. 2007). Importantly, moderate (Blonigen et al. 2005) to relatively strong (Viding et al. 2007) genetic correlations were found.

The etiological association between CU traits and other phenotypes, including hyperactivity and emotional problems, has been less explored. To our knowledge, no published twin study has specifically examined the etiological association between CU traits and hyperactivity in youth. However, one twin study tested whether symptoms of ADHD were associated with different dimensions of psychopathic traits (including CU traits) in adolescence (Forsman et al. 2007). A modest phenotypic correlation was observed between CU traits and symptoms of ADHD, but the authors did not examine whether this association was explained by genetic, shared or non-shared environmental factors. The genetic association between CU traits (more specifically fearless dominance, which encompasses interpersonal-affective traits such as fearlessness and social potency) and emotional problems (i.e., major depression, social phobia and simple phobia) was examined in a study of 17-year-old twins (Blonigen et al. 2005). This study revealed that fearless dominance exhibited a moderate negative genetic correlation with emotional problems, indicating that the same genetic factors that contributed to fearless dominance traits also contributed to *reduced* levels of emotional problems.

In sum, previous research showed that CU traits in youth are under the influence of moderate to strong heritability and that a modest to strong proportion of the factors explaining the genetic variance of conduct problems also explains the genetic variance of CU traits. The degree of etiological association between CU traits and other phenotypes, more specifically hyperactivity and emotional problems, has been less explored. To address these limitations, the current study, employing a twin model-fitting approach, aimed to 1) replicate findings on the etiology of CU traits and their etiological association with conduct problems in a sample of adolescent twins screened for neurodevelopmental problems, and 2) extend research by examining further the etiological associations between CU traits and symptoms of hyperactivity and emotional problems separately. Findings from the current study have the potential to clarify the underlying etiological bases of CU traits and their associations with other psychopathological symptoms.

## Method

### Participants

The participants were drawn from the Child and Adolescents Twin Study in Sweden (CATSS), an ongoing longitudinal twin study targeting all twins born in Sweden since July 1, 1992 (Anckarsäter et al. 2011). As of January 2010, the CATSS included approximately 17,220 twins, representing roughly 80% of all twins born in Sweden since July 1992 (Anckarsäter et al. 2011). All CATSS twins were screened at age 9 or 12 for different neurodevelopmental problems and other mental health problems, including ADHD, autism spectrum disorders, oppositional defiant disorder, conduct disorder, obsessive compulsive disorder, mood disorders and eating problems through the use of the Autism – Tics, ADHD and Other Comorbidities Inventory (A-TAC). This instrument is designed to evaluate all major clinical diagnostic criteria in child and adolescent psychiatry based on the DSM (Anckarsäter et al. 2011; Larson et al. 2013). It has been found to be a reliable and valid screening instrument for childhood neurodevelopmental disorders (Hansson et al. 2005; Larson et al. 2013).

The twins who were born in 1993, 1994 and 1995 were eligible for the follow-up assessment at age 15 (Larson et al. 2013). Those born between 1993 and June 1995 had been screened with the A-TAC at age 12, whereas those born between July and December 1995 had been screened at age 9. A sample of 15-year-olds was selected to create a study group enriched for neurodevelopmental problems, namely the CATSS-15/DOGSS (Developmental Outcomes in a Genetic twin Study in Sweden). This subsample was created to clinically assess the outcomes of the children who had screened positive for neurodevelopmental problems at 9/12 years old using the A-TAC. Same-sex twin pairs in whom at least one of the siblings had screened positive at 9/12 years old for autism spectrum disorders, ADHD, tic disorders, learning disorders, developmental coordination disorder or other mental health disorders with known neurodevelopmental comorbidities (obsessive compulsive disorder, oppositional defiant disorder, conduct disorder, and/or eating disorder) were invited to participate in the follow-up assessment (about 15% of the total CATSS sample). Additionally, a number of randomly selected controls were included (5% of the total CATSS sample). From the 1995 cohort, the inclusion criteria were narrowed, such that only autism spectrum disorders and ADHD were considered (Anckarsäter et al. 2011).

A total of 860 youths were eligible participants based on the above criteria. From these youths, about half consented to participate to the follow-up assessment at age 15, for a total of 451 same-sex twins: 247 screen-positive twins, 155 screen-negative co-twins and 49 randomly selected control twin participants matched for sex and age. From the 247 twins who were screen-positive at 9/12 years old, 198 were for neurodevelopmental problems (i.e., autism spectrum disorders, ADHD, tic disorders, learning disorders, developmental coordination disorder) and 49 were for other mental health problems only (i.e., obsessive compulsive disorder, oppositional defiant disorder, conduct disorder, and/or eating disorder, with no overlap with neurodevelopmental problems; see Larson et al. 2013). Approximately 70% of the screen-positive twins at 9/12 years old, as well as 41% of the screen-negative co-twins and 39% of the controls met DSM diagnostic criteria for developmental disorders and/or other mental health issues (e.g., anxiety, depression, manic disorders) at age 15 based on the Schedule for Affective Disorders and Schizophrenia for School-Age Children (K-SADS; Kaufman et al. 2000). Table 1 provides information about the distribution of the diagnoses at ages 9/12 and 15 years.

**Table 1:** Distribution of the diagnoses at ages 9/12 and 15 years

	Age 9/12^d^		Age 15	
	*n*	%	*n*	%
All neurodevelopmental disorders^a,b^	198	44.0	160	35.5
All other mental health problems only^c^	49	10.9	87	19.3
Specific neurodevelopmental disorders or mental health problems				
Autism spectrum disorders	27	6.0	20	4.4
ADHD	95	21.1	96	21.3
Learning disorders	74	16.4	27	6.0
Tic disorder	35	7.8	64	14.2
Developmental coordination disorder^b^	38	8.4	–	–
Conduct disorder	4	0.9	9	2.0
Oppositional defiant disorder	47	10.4	21	4.7

^a^Neurodevelopmental disorders were defined as autism spectrum disorders and/or ADHD and/or learning disorders and/or tic disorder and/or developmental coordination disorder, with a possible overlap of other mental health problems (Larson et al. 2013)

^b^Developmental coordination disorder had no corresponding diagnosis in the clinical assessment at age 15 years (Larson et al. 2013)

^c^At age 9/12, all other mental health problems were defined as obsessive compulsive disorder and/or oppositional defiant disorder and/or conduct disorder and/or eating disorder with no overlap with neurodevelopmental disorders. At age 15, they additionally included depression, anxiety, stress disorder, mania and/or psychosis (Larson et al. 2013)

^d^One sibling was not screened at age 9/12 years

Twins diagnosed with epilepsy, brain damage, chromosomal aberrations or intellectual disability (*n* = 25) were excluded from the analyses. The final sample included 426 twins aged 15 years (204 complete pairs, of which 24 were control participants, and 18 incomplete pairs, of which 1 was a control participant). In our sample, 38% of mothers and 21% of fathers had earned a university degree. The majority of parents were born in Sweden (93% for mothers, 90% for fathers). The sample consisted of 43.4% monozygotic (MZ) twins (*n* = 185 twins; 79 female) and 56.6% dizygotic (DZ) twins (*n* = 241; 97 female). Zygosity was established through DNA analysis. For twins without DNA samples, an algorithm based on five questions on twin similarity was used. Twins were only assigned zygosity through the algorithm method if the test achieved a 95% probability of producing a correct categorization (Anckarsäter et al. 2011). At age 15, twins involved in the CATSS-15/DOGSS completed a clinical assessment, including the K-SADS (Kaufman et al. 2000) and a number of self-report questionnaires on a variety of physical and mental health subjects, including some that were not evaluated in the wider CATSS study (e.g., callous-unemotional traits) (see Anckarsäter et al. 2011, for more details about the procedures).

### Measures

#### CU Traits

Self-reported CU traits were assessed using the CU dimension scale of the Youth Psychopathic traits Inventory (YPI; Andershed et al. 2002), administered in its original Swedish version. The CU dimension scale includes 15 items assessing callousness (e.g., “*When other people have problems, it is often their own fault, therefore, one should not help them*”), unemotionality (e.g., “*I usually feel calm when other people are scared*”) and remorselessness *(*e.g.*,* “*I seldom regret things I do, even if other people feel that they are wrong*”) (Andershed et al. 2002). Participants rated the degree to which the item applied to them using a 4-point scale with responses ranging from “*Does not apply at all*” to “*Applies very well*” (Andershed et al. 2002). This scale showed good reliability, with an ordinal alpha of .85 (Gadermann et al. 2012). The YPI has demonstrated good construct validity and correlates moderately to strongly with other self-report measures of psychopathy and callous-unemotional traits in various samples of adolescents (Neumann and Pardini 2014; Poythress et al. 2006).

#### Conduct Problems, Hyperactivity and Emotional Problems

Self-reported conduct problems (5 items, e.g., “*I fight a lot. I can make other people do what I want”*), hyperactivity (5 items, e.g., “*I am constantly fidgeting or squirming*”) and emotional problems (5 items, e.g., “*I worry a lot*”) were assessed using the self-report version of the Strengths and Difficulties Questionnaire (SDQ; Goodman et al. 1998). Participants marked each item using a 3-point scale (“*Not true,” “Somewhat true”* or *“Certainly true”*) (Goodman et al. 1998). Ordinal alphas for conduct problems, hyperactivity and emotional problems were .67, .82 and .82, respectively. The SDQ is an established screening instrument and it has been found that high scores on the SDQ are likely to strongly indicate the presence of psychiatric disorder(s) as diagnosed by a clinician (Goodman 2001). Satisfactory internal consistency (Malmberg et al. 2003) and construct validity (Smedje et al. 1999) have been reported for the Swedish version of the SDQ.

### Data Analyses

We used a twin model-fitting approach in the current study. Twin studies rely on the comparison between intra-pair correlations in MZ twins (who are genetically identical) and in DZ twins (who on average share only half of their genes). From this comparison, sources of variability of a phenotype can be estimated in terms of latent genetic effects (A), latent shared environmental effects (C; i.e., environmental influences that make family members similar to each other, which might be, for e.g., socioeconomic status and attitudes within families) and latent non-shared environmental effects (E; i.e., environmental influences that make family members different from each other, which might be, e.g., sibling differences in peer groups and differential parental treatment). When twins are reared together, it is assumed that MZ and DZ twins are equally similar in terms of their environment. When MZ twins are more similar to each other than DZ twins, it can be inferred that this difference is due to genetic effects. Any resemblance between MZ twins not due to genetic effects is attributed to shared environmental effects. Differences between MZ twins are due to their non-shared environmental contributions (Rijsdijk and Sham 2002). The E factor also includes measurement error that may have decreased the similarity between members of a twin pair, such as slightly different testing conditions. This error may lead to a possible overestimation of non-shared environmental effects and therefore, an underestimation of the genetic and shared environmental effects (Brendgen et al. 2012; Neale and Cardon 1992).

**Fig. 1:**
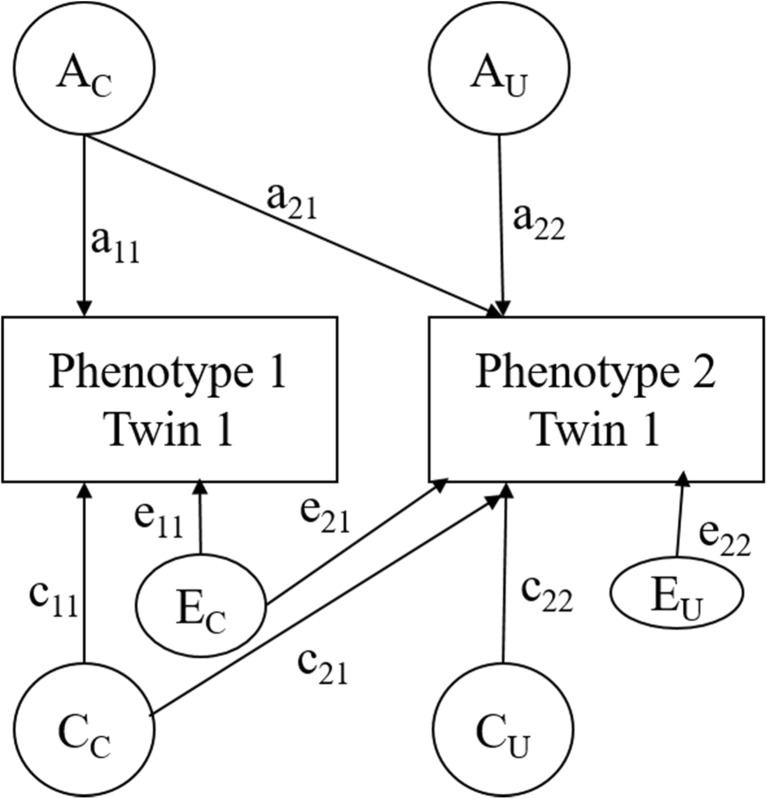
Overview of the bivariate Cholesky model representing common (A_C_, C_C_, E_C_) and unique (A_U_, C_U_, E_U_) latent factors used to evaluate the etiological associations between two phenotypes

Our aim was to evaluate the degree of etiological association between CU traits and other phenotypes separately (i.e., conduct problems, hyperactivity and emotional problems). To this end, we applied a bivariate Cholesky twin model (see Fig. 1) to each pair of phenotypes. Cholesky decompositions parse etiological contributions into common latent factors (A_C_, C_C_, E_C_), which affect both phenotypes, and unique latent factors (A_U_, C_U_, E_U_), which are specific to the second phenotype (Brendgen et al. 2009). As seen in Fig. 1, the a_11_, c_11_ and e_11_ paths are associated with parameters that represent the genetic, shared environmental and non-shared environmental factor loadings of the first phenotype (i.e., CU traits) on the *common* latent factors; a_21_, c_21_ and e_21_ represent the factor loadings of the second phenotype on the *common* latent factors; and a_22_, c_22_ and e_22_ parameters the factor loadings of the second phenotype on the *unique* latent factors (Brendgen et al. 2009).

Using these parameters, we calculated the etiological contributions (%A, %C, %E) specific to each phenotype using model-fitting analyses standard to twin methodology. Next, Cholesky decompositions allowed us to estimate the degree of etiological association between two phenotypes, resulting in a genetic correlation (*r*_*A*_*, r*_*C*_*, r*_*E*_*)*. By squaring this correlation, we obtained the proportion of genetic, shared environmental and non-shared environmental variance common to both phenotypes for all three pairings; e.g., *r*_A_^2^ represented the proportion of genetic variance of the second phenotype explained by genetic factors that *also* influenced the first phenotype (i.e., CU traits).

In order to ensure that our bivariate models were consistent with the data, we estimated a full Cholesky model (i.e., ACE – ACE) for each of the pairings. Next, we evaluated the fit of a number of nested models (e.g., AE – AE), which were obtained by reducing certain factor loadings to 0 (Brendgen et al. 2012). We assessed the fit of each of these models using a maximum-likelihood function (Neale and Cardon 1992). This function provides a goodness-of-fit statistic represented by a likelihood ratio chi-square (χ^2^). A non-significant χ^2^ value (*p* > 0.05) is one indication that the model fits the data and can be used. The selection of best-fitting model was based on the model with the lowest Bayesian information criterion (BIC) (Raftery 1995; Schwarz 1978). We used full information maximum likelihood to include incomplete twin pairs in our model. All analyses were performed using Mplus (Version 7.31; Muthén & Muthén, 1998-2012).

## Results

### Descriptive Statistics

Descriptive statistics for all 426 participants are displayed in Table 2. Before performing the analyses, square root transformations were applied to the conduct problems and emotional problems scales to correct for skewness and kurtosis.

**Table 2:** Characteristics of the twins at age 15 years

	%	*n*	*M*	*SD*	Range	Skewness	Kurtosis
MZ	43.4	185					
Boys	58.7	250					
CU traits		407	13.29	6.53	0–35.00	0.48	−0.12
Conduct problems		414	1.78	1.43	0–9.00	1.25	2.75
Hyperactivity		413	3.54	2.39	0–10.00	0.38	−0.60
Emotional problems		414	2.22	2.09	0–10.00	0.96	0.58

*n* = 426 twins, *MZ* monozygotic twins, *CU* callous-unemotional

### Phenotypic Correlations

The phenotypic correlations between the phenotypes are displayed in Table 3. As expected, CU traits were positively correlated with conduct problems (*r =* .28, *p* < 0.001) and hyperactivity (*r =* .15, *p* < 0.01), and negatively correlated with emotional problems (*r* = −.17, *p* < 0.01).

**Table 3:** Phenotypic correlations between CU traits, conduct problems, hyperactivity and emotional problems

	1	2	3	4
1. CU traits	–			
2. Conduct problems	0.28 (0.18; 0.38)	–		
3. Hyperactivity	0.15 (0.05; 0.25)	0.51 (0.44; 0.59)	–	
4. Emotional problems	−0.17 (−0.27; −0.06)	0.19 (0.09; 0.29)	0.27 (0.18; 0.36)	–

*n* = 221–222 pairs, *CU* callous-unemotional

Confidence intervals (based on 10,000 bootstrap samples) are presented in parentheses

### Intra-Pair Correlations

Intra-pair correlations for CU traits, conduct problems, hyperactivity and emotional problems are shown in Table 4. Importantly, all the MZ correlations were higher than the DZ correlations, indicating evidence for genetic contributions to all four phenotypes.

**Table 4:** Intra-pair correlations for CU traits, conduct problems, hyperactivity and emotional problems

	MZ	DZ
CU traits	0.64 (0.52; 0.76)	0.31 (0.13; 0.48)
Conduct problems	0.41 (0.23; 0.60)	0.29 (0.13; 0.46)
Hyperactivity	0.43 (0.26; 0.60)	0.07 (−0.11; 0.26)
Emotional problems	0.53 (0.38; 0.69)	0.31 (0.14; 0.47)

*n* = 221–222 pairs; *CU* callous-unemotional, *MZ* monozygotic twins, *DZ* dizygotic twins

Confidence intervals (based on 10,000 bootstrap samples) are presented in parentheses

### Bivariate Analyses

We performed three sets of Cholesky bivariate decompositions: (1) CU traits – conduct problems, (2) CU traits – hyperactivity and (3) CU traits – emotional problems. As displayed in Table 5, fit indices indicated that the AE – ACE model best fit the data for the etiological association between CU traits and conduct problems, whereas the AE – AE model best fit the data for the etiological association between CU traits and hyperactivity as well as between CU traits and emotional problems.

**Table 5:** Model fitting results of bivariate analysis of CU traits and conduct problems, CU traits and hyperactivity, and CU traits and emotional problems

	Model information				Log likelihood ratio test	
	LL(*df)*	χ^2^	*p*	BIC	Δχ^2^(*df)*	*p*
*CU traits – conduct problems*						
ACE x ACE	−1683.34(5)	2.15	0.829	3426.12		
ACE x AE	−1683.92(7)	3.29	0.857	3416.46	1.14(2)	0.564
**AE x ACE**	**−1683.37(7)**	**2.19**	**0.949**	**3409.99**	**0.04(2)**	**0.978**
AE x AE	−1683.93(8)	3.32	0.913	3411.08	1.17(3)	0.760
*CU traits – hyperactivity*						
ACE x ACE	−2243.69(5)	5.24	0.388	4546.80		
ACE x AE	−2243.78(7)	5.43	0.608	4536.19	0.19(2)	0.908
AE x ACE	−2243.81(7)	5.49	0.600	4536.25	0.26(2)	0.880
**AE x AE**	**−2243.81(8)**	**5.49**	**0.704**	**4530.85**	**0.26(3)**	**0.968**
*CU traits – emotional problems*						
ACE x ACE	−1808.69(5)	5.43	0.366	3676.81		
ACE x AE	−1809.60(7)	7.25	0.403	3667.82	1.82(2)	0.402
AE x ACE	−1809.52(7)	7.09	0.419	3667.66	1.66(2)	0.436
**AE x AE**	**−1809.60(8)**	**7.25**	**0.510**	**3662.42**	**1.82(3)**	**0.610**

*CU* callous-unemotional, *LL* log likelihood, *df* degrees of freedom, *BIC* Bayesian information criterion

The best fitting models are in bold

The bivariate parameter estimates associated with the best fitting models are displayed in Table 6. We used these to estimate the individual etiology of each phenotype. For instance, the variance in CU traits was largely explained by genetic factors (63%), although there was evidence of a moderate non-shared environmental contribution (37%). The variance in conduct problems was explained by genetic (22%), shared environmental (19%), and non-shared environmental factors (59%). The variance in hyperactivity was explained by genetic (37%) and non-shared environmental (63%) factors. Lastly, the variance in emotional problems was divided into genetic (53%) and non-shared environmental (47%) contributions. We found no evidence of shared environmental contributions to the variance in CU traits, hyperactivity and emotional problems.

**Table 6:** Bivariate model parameters

	A	C	E	%A	%C	%E
*CU traits – conduct problems (AE x ACE)*						
CU traits^a^	5.16 (4.46; 5.81)	–	3.97 (3.27; 4.62)	62.9	–	37.1
Conduct problems				22.3	18.7	59.1
Common effects^b^	0.23 (0.14; 0.31)	–	−0.01 (−0.10; 0.09)			
Unique effects^c^	0.19 (0.00; 0.44)	0.27 (0.00; 0.37)	−0.49 (−0.55; −0.39)			
*CU traits – hyperactivity (AE x AE)*						
CU traits^a^	5.19 (4.48; 5.86)	–	3.94 (3.25; 4.59)			
Hyperactivity				36.7	–	63.3
Common effects^b^	0.52 (0.15; 0.90)	–	−0.07 (−0.43; 0.28)			
Unique effects^c^	1.34 (0.86; 1.67)	–	1.89 (1.64, 2.11)			
*CU traits – emotional problems (AE X AE)*						
CU traits^a^	5.16 (4.45, 5.82)	–	3.96 (3.27, 4.60)			
Emotional problems				52.9	–	47.1
Common effects^b^	−0.14 (−0.28, −0.01)	–	−0.03 (−0.15, 0.09)			
Unique effects^c^	0.60 (0.49, 0.68)	–	0.58 (0.49, 0.66)			

*CU* Callous-unemotional, *A* genetic effects, *C* shared environment effects, *E* non-shared environment effects

^a^associated paths: a_11_, c_11_, e_11_; ^b^associated paths: a_21_, c_21_, e_21_; ^c^associated paths: a_22_, c_22_, e_22_

Confidence intervals (based on 10,000 bootstrap samples) are presented in parentheses

We calculated the etiological correlations between each bivariate pairing (see Table 7). A strong genetic correlation was found between CU traits and conduct problems (*r*_A_ = 0.77 [0.37; 1.00]). This indicates that 59% (0.77^2^) of the genetic variance of conduct problems was explained by factors that also explained the genetic variance of CU traits. A relatively moderate genetic correlation was found between CU traits and hyperactivity (*r*_A_ = 0.36 [0.11; 0.66]), indicating that 13% (0.36^2^) of the genetic variance of hyperactivity was explained by genetic factors that also influenced CU traits. Lastly, a relatively modest, albeit significant, negative genetic correlation was found between CU traits and emotional problems (*r*_A_ = −0.23 [−0.44; −0.01]). This suggests that 5% (−0.23^2^) of the genetic variance of emotional problems was explained by factors that also explained the genetic variance of CU traits. Weak and non-significant non-shared environmental correlations were also found for each model.

**Table 7:** Etiological correlations between CU traits and symptoms of conduct problems, hyperactivity and emotional problems

	CU traits		
	*r*_A_ (95% CI)	*r*_C_ (95% CI)	*r*_E_ (95% CI)
Conduct problems	**0.77** (0.37; 1.00)	–	−0.02 (−0.21; 0.19)
Hyperactivity	**0.36** (0.11; 0.66)	–	−0.04 (−0.23; 0.15)
Emotional problems	**−0.23** (−0.44; −0.01)	–	−0.05 (−0.25; 0.15)

*CU* callous-unemotional, *CI* confidence intervals (based on 10,000 bootstrap samples)

A parameter is statistically significant if the CI does not include 0. A 95% CI indicates a 95% probability of the data being correctly classified

Significant correlations are in bold

## Discussion

In this study, we examined the etiological associations between CU traits and symptoms of conduct problems, hyperactivity and emotional problems through the use of a twin design. This allowed us 1) to replicate findings on the relatively high heritability of CU traits and the genetic correlation between CU traits and conduct problems in a sample of adolescent twins screened for neurodevelopmental problems, and 2) to investigate further the etiological associations between CU traits and symptoms of hyperactivity and emotional problems separately. Findings from this study extend research in three main respects.

First, we found substantial genetic contributions to CU traits and a strong genetic correlation between CU traits and conduct problems. These findings are consistent with previous research examining the etiological association between CU traits and conduct problems in youth (Bezdjian et al. 2011; Viding et al. 2007). However, unlike previous studies (Bezdjian et al. 2011; Viding et al. 2007), we did not find a significant non-shared environmental correlation between CU traits and conduct problems. The substantial genetic correlation suggests that future molecular genetic research should focus on the identification of common genes that contribute to CU traits and conduct problems (Viding et al. 2007). Still, the non-overlapping genetic variance suggests some independence in the underlying biological mechanisms leading to the development of CU traits and conduct problems (Taylor et al. 2003; Viding et al. 2007). For instance, biological mechanisms related to temperamental characteristics (e.g., fearlessness; Glenn et al. 2007; Waller et al. 2016), emotion regulation and empathy could be represented in the residual genetic variance found for CU traits (Taylor et al. 2003).

Second, to our knowledge, this is the first published study to examine the etiological association between CU traits and hyperactivity in youth. Although past research showed that the two phenotypes often co-occur (Fontaine et al. 2008; Fontaine et al. 2011; Frick et al. 2003a; Nagin and Tremblay 2001), our findings suggest that they share genetic etiological factors, but only to some extent. Indeed, unlike CU traits and conduct problems, the genetic correlation between CU traits and hyperactivity was not strong. The substantial non-overlapping genetic variance suggests important independence in the underlying biological mechanisms leading to the development of CU traits and hyperactivity, which appears to be in line with past research suggesting that hyperactivity is not a significant risk factor associated with the development of CU/psychopathic traits (Barry et al. 2000; Forsman et al. 2007). Biological mechanisms related to temperamental characteristics (e.g., fearlessness), emotional regulation and empathy could be represented in the residual variance found for CU traits, but to a greater extent than when examining the etiological overlap between CU traits and conduct problems. Future research is needed to further our understanding about the etiological associations between these phenotypes.

Third, we found a relatively modest, but significant negative genetic correlation between CU traits and emotional problems, which is in line with the findings reported by Blonigen et al. (2005). In this previous study, a negative genetic correlation was reported between fearless dominance, which covered a wide range of interpersonal-affective traits associated with psychopathy, and emotional problems, which included symptoms of phobia and depression. Because we focused on CU traits, instead of a wider range of interpersonal-affective traits, the current study extends previous findings by increasing the level of specificity in the examination of the etiological association between psychopathic traits and emotional problems. The negative genetic correlation between CU traits and emotional problems suggests that the genetic factors influencing the *increase* of one phenotype contribute to the *decrease* of the other. This could suggest that CU traits may act as a protective factor for emotional problems (or that emotional problems may act as a protective factor for CU traits). It could be that the biological mechanisms associated with fearlessness in CU traits (Glenn et al. 2007; Waller et al. 2016) provide a resiliency to developing a broad range of emotional problems, such as anxiety and depression symptoms (Blonigen et al. 2005). Still, the relatively modest negative genetic correlation between the two phenotypes suggests important independence in their respective underlying biological mechanisms.

There are a number of strengths to this study, including the use of a measure of CU traits over a broader measure of psychopathic traits, which increases the specificity of our findings. Moreover, to our knowledge, this was the first published study to explore the etiological association between CU traits and hyperactivity in youth. However, this study has a number of limitations. First, we were unable to conduct the analyses separately for boys and girls due to our sample size. Taking sex differences into account may be important because boys tend to have higher scores than girls on CU traits, conduct problems, and hyperactivity, but lower scores on emotional problems (Essau et al. 2006; Fontaine et al. 2008; Lewinsohn, Gotlib, Lewinsohn, Seeley, & Allen 1998). In addition, sex differences in the etiology of the behaviours and traits at study have been previously reported. For instance, lower heritability and higher shared environmental contributions for CU traits (Fontaine et al. 2010; Viding et al. 2007) as well as lower shared and non-shared environmental correlations between CU traits and conduct problems have been found in girls (Viding et al. 2007). Second, the sample size may have influenced the results. More specifically, the non-significant findings for environmental influences could potentially be related to the relative small sample size and limited statistical power.

Third, the internal consistency of the measure of conduct problems was moderate (α = .67). This may have produced relatively lower heritability estimates for our measure of conduct problems and a more conservative estimate of the magnitude of the association between CU traits and conduct problems. Indeed, in samples of adolescents, higher estimates of genetic influences to conduct problems based on the parent-report version of the SDQ have been reported (e.g., a^2^ = .73; Pingault et al. 2015). However, estimates of genetic influences to conduct problems based on the self-report version of the SDQ tend to be lower than for parent-reports (e.g., a^2^ = .35 for self-reports and a^2^ = .54 for parent-reports; Scourfield et al. 2004). Fourth, we used the emotional problems scale of the SDQ, which is a brief screening instrument (Goodman 2001). Replications of our findings using more comprehensive measures of different types of emotional problems (e.g., anxiety and depression separately) are needed.

Fifth, because all the measures were based on the youths’ reports, there is a possibility that our findings were partly influenced by shared method variance. Finally, as in all twin studies, it must be noted that the non-shared environment influences can be affected by unsystematic, chance events that, when compounded over time, make twins different in unpredictable ways (Plomin and Daniels 1987). This makes non-shared environmental influences difficult to interpret. However, our study mainly focuses on the significant genetic correlations, and as such, this prospect does not appear to overly affect our findings or our conclusions. Finally, the CATSS-15/DOGSS is mainly composed of children who screened positive for neurodevelopmental problems/mental health problems or at-risk children (i.e., screen-negative children who are considered to be genetically at-risk siblings). This provided a unique opportunity to replicate and extend previous findings based on population-based twin samples to a sample composed of clinical and at-risk children identified from a population-based sample of twins. Replications involving youth from various backgrounds are needed to increase the generalizability of the findings. Although replications of our findings using larger twin samples would be crucial, our study is a step further toward a better understanding of the etiological overlap between CU traits and other symptoms of psychopathology.

This study raises a number of implications. It is important to note that genetic vulnerability does not mean immutability. Genetically-influenced behaviours can be buffered by preventive and treatment strategies, which could be considered as positive gene-environment interactions (Fontaine et al. 2018). We found a genetic correlation between CU traits and conduct problems, but contrary to previous studies (Bezdjian et al. 2011; Viding et al. 2007), we did not find a significant non-shared environmental correlation. Given the past and the current findings, future research is needed to identify measured environmental factors (e.g., parenting behaviours) that may be common or specific to CU traits and related symptoms of psychopathology, which in turn could be targeted in the context of intervention programs. Investigations focusing on child-specific environmental factors within twin designs may be particularly promising.

In sum, we found a strong positive genetic correlation between CU traits and conduct problems, a relatively moderate genetic correlation between CU traits and hyperactivity, and a modest negative genetic correlation between CU traits and emotional problems. Studies based on prospective longitudinal data would be needed to advance further our understanding of the potential etiological mechanisms underlying the development of CU traits and related psychopathological symptoms.
